# Genome-Based Metabolic Reconstruction Unravels the Key Role of B12 in Methionine Auxotrophy of an *Ortho*-Phenylphenol-Degrading *Sphingomonas haloaromaticamans*

**DOI:** 10.3389/fmicb.2019.03009

**Published:** 2020-01-10

**Authors:** Chiara Perruchon, Sotirios Vasileiadis, Evangelia S. Papadopoulou, Dimitrios G. Karpouzas

**Affiliations:** Laboratory of Plant and Environmental Biotechnology, Department of Biochemistry and Biotechnology, University of Thessaly, Larissa, Greece

**Keywords:** *Sphingomonas haloaromaticamans*, methionine, *ortho*-phenylphenol, genome-based metabolic reconstruction, B12 auxotrophy

## Abstract

Auxotrophy to amino acids and vitamins is a common feature in the bacterial world shaping microbial communities through cross-feeding relations. The amino acid auxotrophy of pollutant-degrading bacteria could hamper their bioremediation potential, however, the underlying mechanisms of auxotrophy remain unexplored. We employed genome sequence-based metabolic reconstruction to identify potential mechanisms driving the amino acid auxotrophy of a *Sphingomonas haloaromaticamans* strain degrading the fungicide *ortho*-phenylphenol (OPP) and provided further verification for the identified mechanisms *via in vitro* bacterial assays. The analysis identified potential gaps in the biosynthesis of isoleucine, phenylalanine and tyrosine, while methionine biosynthesis was potentially effective, relying though in the presence of B12. Supplementation of the bacterium with the four amino acids in all possible combinations rescued its degrading capacity only with methionine. Genome sequence-based metabolic reconstruction and analysis suggested that the bacterium was incapable of *de novo* biosynthesis of B12 (missing genes for the construction of the corrin ring) but carried a complete salvage pathway for corrinoids uptake from the environment, transmembrane transportation and biosynthesis of B12. In line with this the bacterium maintained its degrading capacity and growth when supplied with environmentally relevant B12 concentrations (i.e., 0.1 ng ml^–1^). Using genome-based metabolic reconstruction and *in vitro* testing we unraveled the mechanism driving the auxotrophy of a pesticide-degrading *S. haloaromaticamans*. Further studies will investigate the corrinoids preferences of *S. haloaromaticamans* for optimum growth and OPP degradation.

## Introduction

Auxotrophy is the inability of an organism to synthesize a particular biomolecule which is necessary for its growth. Auxotrophic bacterial mutants are known to evolve spontaneously upon long-term growth in an environment rich in biomolecules leading bacteria to genetic drift and loss of the corresponding biosynthetic pathways (Van der Kaaij et al., 2014). Amino acid auxotrophy is a common feature of bacterial genomes which have been evolutionary optimized to reduce the metabolic burden stemming from the production of energetically costly amino acids (i.e., phenylalanine, tyrosine, and methionine) (Mee et al., 2014). Instead their biosynthesis is assigned to a few bacteria leading to the establishment of a mutualistic trade among bacteria to ensure access to essential biomolecules (D’Souza et al., 2014; Pande et al., 2015). Vitamin auxotrophy constitutes another frequent bacterial phenotype in aquatic ecosystems, terrestrial ecosystems, and the human gut. Comparative genomics showed that each of the eight vitamins was produced by 45–60% of the gut bacteria suggesting that exchange of vitamins amongst human gut bacteria enables the survival of members that could not synthesize any of these co-factors (Magnúsdóttir et al., 2015). Similarly, Gomez-Consarnau et al. (2018) showed that vitamins synthesis and exchange by bacteria leads to a mosaic of metabolic interdependencies which contribute to the shaping of microbial communities in marine ecosystems. For instance, *Rhodobacterales* dominated the expression of vitamin-B12 synthesis, but relied on *Flavobacteria* for the production and supply of vitamin B7. Price et al. (2018) challenged experimentally the value of comparative genomic studies (i.e., their data showed that auxotrophy in bacteria is not as common as claimed by comparative genomic studies) and suggested that their findings should be always verified experimentally.

Amino acids auxotrophy has been reported in several pesticide-degrading bacteria. First, Sørensen et al. (2001) noted that an isoproturon-degrading *Sphingomonas* strain SRS2 was able to maintain its degrading capacity only under external supplementation with casamino acids (CA); a mixture of all 20 essential amino acids. Follow up studies revealed that strain SRS2 required external supply of methionine or the presence of another bacterium in the culture (Sørensen et al., 2002), which was probably able to provide to strain SRS2 the missing nutrient factors. The need for external supply of amino acids to maintain their degrading activity and growth on organic pollutants is widespread among sphingomonads with examples of a *Sphingomonas xenophaga* degrading 1-amino-4-bromoanthraquinone-2-sulfonic acid (Lu et al., 2015), a *Sphingobium* sp. degrading MCPA (Onneby et al., 2014), and a *Sphingomonas* sp. degrading triclosan (Hay et al., 2001). This has been assumed to be the result of incomplete amino acid biosynthetic pathways, however, the exact underlying mechanism remains unknown.

Vitamin B12 (cyanocobalamin) and its methyl and adenosyl derivatives, are essential co-factors of several key enzymes like methionine synthase, ribonucleotide reductase, diol dehydratase, and ethanolamine ammonia lyase (Rodionov et al., 2003). Its biosynthesis is the most energetically costly pathway and is restricted to a number of bacteria and archaea (Roth et al., 1996). A comparative genomic analysis showed that nearly 90% of the 11,000 bacterial genomes examined had at least one and max 15 B12-dependent enzymatic families, yet only 37% carried the full B12 biosynthetic pathway (Shelton et al., 2019), reinforcing the key role of B12 in the synergism among microbial populations. Such B12-based symbiotic relationships have been described between algae and bacteria (Croft et al., 2005; Romine et al., 2017) or between bacteria (Men et al., 2012) shaping microbial communities in marine water systems (Heal et al., 2017) and the human gut (Degnan et al., 2014).

We recently isolated a *Sphingomonas haloaromaticamans* strain which was able to degrade the fungicide *ortho*-phenylphenol (OPP) only when supplemented with CA or co-cultivated with other bacteria (Perruchon et al., 2016). We aimed to further explore the mechanism driving this auxotrophy of the *S. haloaromaticamans* strain to amino acids. The hypothesis initially tested was that *S. haloaromaticamans* has one or more incomplete amino acid biosynthesis pathways, hence the need for external supply of CA. To verify this hypothesis, genome-based metabolic reconstruction of the 20 amino acids biosynthesis in *S. haloaromaticamans* identified possible gaps in the biosynthesis of three amino acids, in addition to methionine whose biosynthetic pathway was complete but relied on the *de novo* biosynthesis of B12. The latter was needed as a co-factor of methionine synthase since the bacterium did not carry a B12-indepenedent isofunctional enzyme. *In vitro* assays identified methionine as the amino acid imposing the auxotrophy of *S. haloaromaticamans* and genome sequence-based metabolic reconstruction suggested that the bacterium was unable to synthesize B12. Further *in vitro* studies with a range of B12 concentrations verified its key role in the auxotrophy of *S. haloaromaticamans*, which could cover its B12 requirements probably by uptake and utilization of B12 through the corrinoids salvage pathway present in its genome.

## Materials and Methods

### Bacterial Strain, Growth Conditions, and Chemicals

The strain *S. haloaromaticamans* used in the current study was isolated from soil of a wastewater disposal site and it was able to degrade the fungicide OPP only when supplemented with CA (Perruchon et al., 2016). The bacterium was routinely cultivated in minimal salts media supplemented with nitrogen (MSMN), OPP (30–50 mg L^–1^) and CA in a shaking incubator at 27°C in the dark. MSMN preparation and OPP chromatographic analysis was as described by Perruchon et al. (2016), while bacterial growth was determined by measurement of the optical density at 600 nm (OD_600_). Inoculation of flasks was performed by fresh bacterial cells grown at the mid-log phase, pelleted by centrifugation, washed three times with sterile ddH_2_O and resuspended with MSMN to an OD_600_ of 0.1.

L-methionine, L-isoleucine, L-tyrosine, L-phenylalanine, L-homoserine, O-succinyl-L-homoserine, L-cystathionine, L-homocysteine and cyanocobalamin (synonym to vitamin B12 in the manuscript), were purchased by Sigma-Aldrich (Taufkirchen, Germany) and they were used for the preparation of aqueous solutions (0.05 mM). These were filter sterilized and used for the preparation of MSMN containing amino acids, B12 and intermediates of methionine biosynthesis at the desired concentrations.

### Bioinformatic and Phylogenetic Analysis

#### Genome Sequence-Based Metabolic Reconstruction of Amino Acids and B12 Biosynthesis in *Sphingomonas haloaromaticamans*

Genomic analysis of *S. haloaromaticamans* has been described by Perruchon et al. (2017) and the assembled and annotated genome is available at DDBJ/ENA/GenBank under the accession number MIPT00000000. The genome annotation data were used for obtaining the Enzyme Commission (EC) numbers and reconstruct the biosynthetic pathways of all amino acids and B12 using the Pathway Tools software v19.0 (Karp et al., 2015) bundled with the EcoCyc (Karp et al., 2014), BioCyc and MetaCyc (Caspi et al., 2016) databases. Missing genes were searched against the genome translated open reading frames (ORFs) after downloading the associated protein sequences of the RefSeq database of the National Center for Biotechnology Information (NCBI) using the basic local alignment search tool (BLAST) v2.7.1+ (Camacho et al., 2009).

#### B12 Transporters and Riboswitches Identification in the Genome of *Sphingomonas haloaromaticamans*

The genome of *S. haloaromaticamans* was searched for TonB-dependent transporters (TBDT) and associated energy transducing ABC transporters involved in the uptake and translocation of corrinoids. Beyond the original annotation described in Perruchon et al. (2017), a second NCBI RefSeq based annotation was performed with BLAST. We further looked for cobalamin-associated riboswitches, acting as regulatory elements of B12 uptake, using the Riboswitch scanner web-based tool (Mukherjee and Sengupta, 2016).

#### Phylogenetic Analysis of MetB/Z

Closely related sequences to MetB/Z found to be encoded in the genome of *S. haloaromaticamans* were retrieved from NCBI with BLASTv2.2.27+ and were clustered with Cdhit v4.6 (Li et al., 2001) to reduce sequence redundancy. The sequences were then aligned with Muscle v3.8.31 (Edgar, 2004). Improperly aligned and uninformative alignment blocks were removed using Gblocks v0.91b (Talavera and Castresana, 2007). The remaining concatenated alignment blocks were subjected to maximum likelihood phylogenies with the RAxML software v8.1.24 (Stamatakis, 2014) and 1000 bootstrap replicates using the best model according to ProtTest v3.4 (Abascal et al., 2005) and the associated Akaike information criterion values. Tree visualization was performed using the APE v3.5 (Paradis et al., 2004) and Phangorn v2.0.4 (Schliep, 2011) R v3.3.1^18^ software packages (R Core Team, 2015).

### Degradation of *Ortho*-Phenylphenol by *Sphingomonas haloaromaticamans* Externally Supplied With Amino Acids

Triplicate 40-ml cultures of MSMN + OPP (50 mg L^–1^) were supplemented with CA (0.15 g L^–1^, CA) or methionine (3.6 mg L^–1^, M), isoleucine (6.75 mg L^–1^, I), tyrosine (2.7 mg L^–1^, T), and phenylalanine (5.7 mg L^–1^, P) added individually and in all possible combinations. These concentrations were selected based on the concentrations of the given amino acids in the CA. Triplicate MSMN + OPP (50 mg L^–1^) samples not supplemented with any amino acids were also included for comparative purposes. All the above cultures were inoculated with *S. haloaromaticamans*as described above. Immediately after inoculation and at regular intervals thereafter aliquots (0.5 ml) of each culture were removed and analyzed for residues of OPP. The degradation of OPP was also measured in triplicate flasks containing MSMN + OPP (50 mg L^–1^) which were not inoculated to serve as abiotic controls.

### Degradation of *Ortho*-Phenylphenol by *Sphingomonas haloaromaticamans* Externally Supplied With B12 and Methionine Precursors

Triplicate 40-ml cultures of MSMN + OPP (30 mg L^–1^) were supplemented with appropriate amounts of aqueous solutions of 0.05 mM of B12, methionine and its precursors homoserine, *O*-succinyl-homoserine, cystathionine and homocysteine. In the case of homocysteine, the ultimate precursor in the biosynthesis of methionine, three further cultures were co-supplemented with B12 to evaluate if its addition rescues the OPP degradation capacity of the bacterium. Triplicate cultures of MSMN + OPP (30 mg L^–1^) not supplemented with methionine, B12 or any methionine precursor were also included for comparative purposes. All these cultures were inoculated with *S. haloaromaticamans* as described above. Immediately after inoculation and at regular intervals thereafter aliquots (0.5 ml) of each culture were removed and analyzed for OPP. The degradation of OPP was also measured in triplicate MSMN + OPP (30 mg L^–1^) not inoculated with the bacterium to serve as abiotic controls.

### Degradation of *Ortho*-Phenylphenol by *Sphingomonas haloaromaticamans* Supplied With a Range of B12 Concentrations

Triplicate 40-ml cultures of MSMN + OPP (25 mg L^–1^) were supplemented with various amounts of different aqueous solutions of B12 (1, 100 and 10000 mg L^–1^) aiming to final concentrations of 0.1, 0.5, 1, 5, 10, 50, 100, 1000, and 10000 ng ml^–1^ in the medium. Triplicate cultures of MSMN + OPP (30 mg L^–1^) not supplemented with B12 were also included for comparative purposes. All cultures were inoculated with *S. haloaromaticamans* as described above. Immediately after inoculation and at regular intervals thereafter aliquots (0.5 ml) of each culture were removed and analyzed for OPP. The degradation of the fungicide was also measured in triplicate MSMN + OPP which were not inoculated to serve as abiotic controls. In parallel, we determined the growth of the bacterium along the degradation of OPP as described above.

## Results and Discussion

### Genome Sequence-Based Metabolic Reconstruction of the Biosynthesis of Amino Acids in *Sphingomonas haloaromaticamans*

**FIGURE 1:**
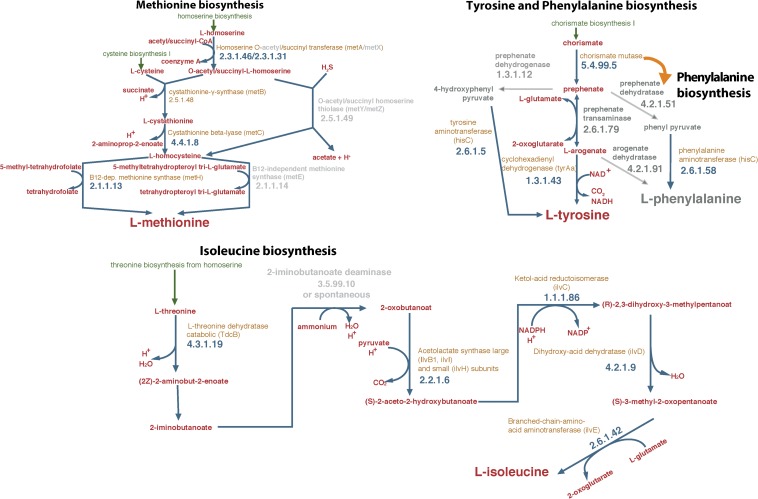
Reconstruction of the biosynthetic pathways of methionine, isoleucine, tyrosine, and phenylalanine of *Sphingomonas haloaromaticamans* based on its genome analysis and assemblage with the Pathway Tools software suit v18.5 (Karp et al., 2015) and the associated database. Gray color arrows indicate the absence of the enzyme from the draft genome of *S. haloaromaticamans*. Gene annotation evidence according to BioCyc and RefSeq database hits is provided in Supplementary Table S1.

The first objective of the study was to identify, based on the draft genome of *S. haloaromaticamans*, biosynthetic pathways of amino acids which were potentially incomplete. We noted possible gaps in the biosynthetic pathways of phenylalanine, tyrosine and isoleucine (Figure 1 and Supplementary Table S1). The biosynthetic pathway of phenylalanine and tyrosine was lacking the enzyme prephenate transaminase (EC 2.6.1.79) (Figure 1). This is commonly found in arogenate-competent bacteria, like α-proteobacteria, and is responsible for the transamination of prephenate to arogenate which is then used for the biosynthesis of tyrosine or phenylalanine (Graindorge et al., 2014). Alternatively, the biosynthesis of tyrosine and phenylalanine proceeds via the transformation of prephenate to 4-hydroxyphenyl pyruvate or phenyl pyruvate, which are then transaminated, with glutamate as amino group donor, to tyrosine or phenylalanine, respectively (Ikeda, 2006). Genes for these pathways were not found in the genome of *S. haloaromaticamans*. Instead we identified two copies of the *hisC* gene encoding an imidazole acetole phosphate aminotransferase, which has been reported before in another α-proteobacterium (*Zymomonas mobilis*) to act also as tyrosine transaminase and phenylalanine transaminase (Gu et al., 1995). This might indicate that the tyrosine, phenylalanine biosynthetic pathway is not incomplete in *S. haloaromaticamans*, however, both amino acids were included in our *in vitro* tests for completeness.

No gene encoding 2-iminobutanoate deaminase (EC.3.5.99.10), an enzyme with potential role in the biosynthesis of isoleucine was detected in the draft genome of *S. haloaromaticamans* (Figure 1). The transformation of threonine to 2-oxobutanoate was originally considered an one-step process controlled by threonine ammonia lyase (*tdcB*) (Risso et al., 2008). It is now suggested that this metabolic step is a two-step process involving the formation of two intermediates [(2Z)-2-aminobut-2-enoate and 2-iminobutanoate] before final formation of 2-oxobutanoate. Lambreht et al. (2012) proposed that 2-iminobutanoate deaminase accelerates the transformation of 2-iminobutanoate to 2-oxobutanoate, a reaction which could occur spontaneously but its acceleration could protect cells from the reactive intermediates. This indicates that isoleucine biosynthesis in *S. haloaromaticamans* might not be incomplete but it was included in the following *in vitro* assays for completeness.

In contrast to the above three amino acids, the genome of *S. haloaromaticamans* carries the necessary genes for the biosynthesis of methionine (Figure 1 and Supplementary Table S1). However, functional annotation of genes in the pathway required clarification. We detected *metA* encoding homoserine-*O*-succinyl transferase (EC 2.3.1.46), which drives the transformation of homoserine to *O*-succinyl-homoserine. This is transformed to homocysteine *via* transsulfuration or sulfhydrylation, depending on the S source, cysteine, or sulfide, respectively (Hwang et al., 2002). The former is a two-step process involving *metB* and *metC* encoding cystathionine-γ-synthase (EC 2.5.1.48) and cystathionine-β-lyase (EC 4.4.1.8), respectively. The latter is controlled by *O*-succinyl homoserine sulfhydrylase (EC 4.2.99.9) encoded by *metZ* (Ferla and Patrick, 2014). A gene showing high homology both to *metB* and *metZ* was detected in the genome of *S. haloaromaticamans*. The enzymes encoded by these two genes belong to the same cystathionine gamma-synthase (CGS) evolutionary family (Gophna et al., 2005) and could function either as cystathione-γ-synthase or as *O*-succinyl homoserine sulfhydrylase depending on the source of S (Hacham et al., 2003). Phylogenetic analysis showed that the relevant gene clustered with *metB* genes of various *Sphingomonas* strains (Supplementary Figure S1) indicating a probable function as cystathionine-γ-synthase. The final step of methionine biosynthesis, the transformation of homocysteine to methionine, is controlled by two isoforms of methionine synthase; a cobalamin-independent (MetE; 5-methyltetrahydropteroyltri-L-glutamate:L-homocysteine S-methyltransferase, EC.2.1.1.14) and its cobalamin-dependent isoform (MetH, EC.2.1.1.13) (Gonzalez et al., 1996), only the latter detected in the genome of *S. haloaromaticamans*. In most bacteria, *metE* and *metH* coexists, however, several bacteria possess only one of these two genes (Shelton et al., 2019). The widespread B12 auxotrophy of algae has been attributed to the evolutionary loss of MetE (Helliwell et al., 2011). Hence, the presence of only *metH* in the genome of *S. haloaromaticamans* might be a limiting factor in the biosynthesis of methionine, for this reason it was included in our *in vitro* studies.

### Methionine External Supply Rescues the Degradation Phenotype of *Sphingomonas haloaromaticamans*

**FIGURE 2:**
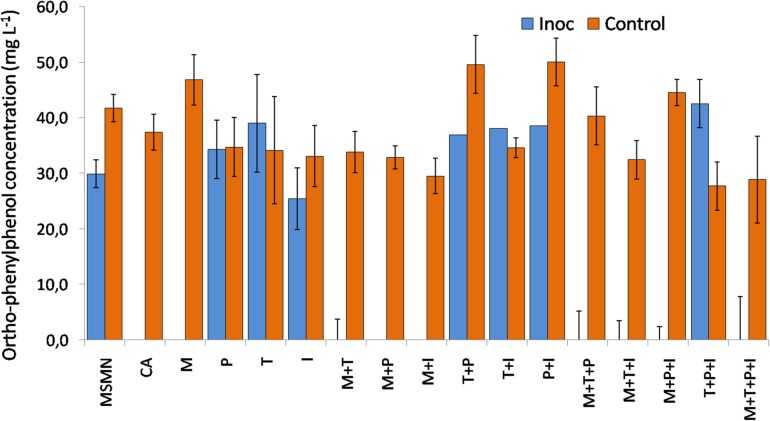
Degradation of OPP 48 h after inoculation (Inoc) with *S. haloaromaticamans* in duplicate cultures of a selective minimal medium (MSMN) supplemented with CA (0.15 g L^–1^, CA) or methionine (3.6 mg L^–1^, M), isoleucine (6.75 mg L^–1^, I), tyrosine (2.7 mg L^–1^, T), and phenylalanine (5.7 mg L^–1^, P) added individually and in all possible combinations. The degradation of OPP was also determined in duplicate inoculated cultures of MSMN without any amino acid supplementation (MSMN) and in corresponding non-inoculated controls (Control).

*Sphingomonas haloaromaticamans* was able to effectively degrade OPP in 7 days in the presence of CA and in all treatments where methionine was provided either alone or in combination with other amino acids (Figure 2). In contrast, limited or no degradation of OPP was observed in the non-inoculated cultures and in the *S. haloaromaticamans* cultures supplemented with the other three amino acids and their combinations. Methionine is the less abundant amino acid in bacterial proteins (Pasamontes and Garcia-Vallve, 2006) but plays an important role in the initiation of translation, has a structural role in hydrophobic cores of proteins, is involved in stabilizing interactions with aromatic amino acids in 1/3 of all known protein structures (Valley et al., 2012), and it is the key component of the cofactor S-adenosyl-methionine which constitutes the main cellular carrier of methyl groups (Chiang et al., 1996). Our findings are in agreement with previous studies which have reported an auxotrophy of other pesticide-degrading sphingomonads to methionine (Sørensen et al., 2002; Onneby et al., 2014). However, none of the above studies looked further into the mechanism driving the reported auxotrophy. In light of the genome-based metabolic analysis of the methionine pathway the observed auxotrophy of *S. haloaromaticamans* to methionine could be the result of the absence of a complete B12 biosynthetic pathway in the studied bacterium.

### Genome Sequence-Based Metabolic Reconstruction of the Biosynthesis of B12 in *Sphingomonas haloaromaticamans*

**FIGURE 3:**
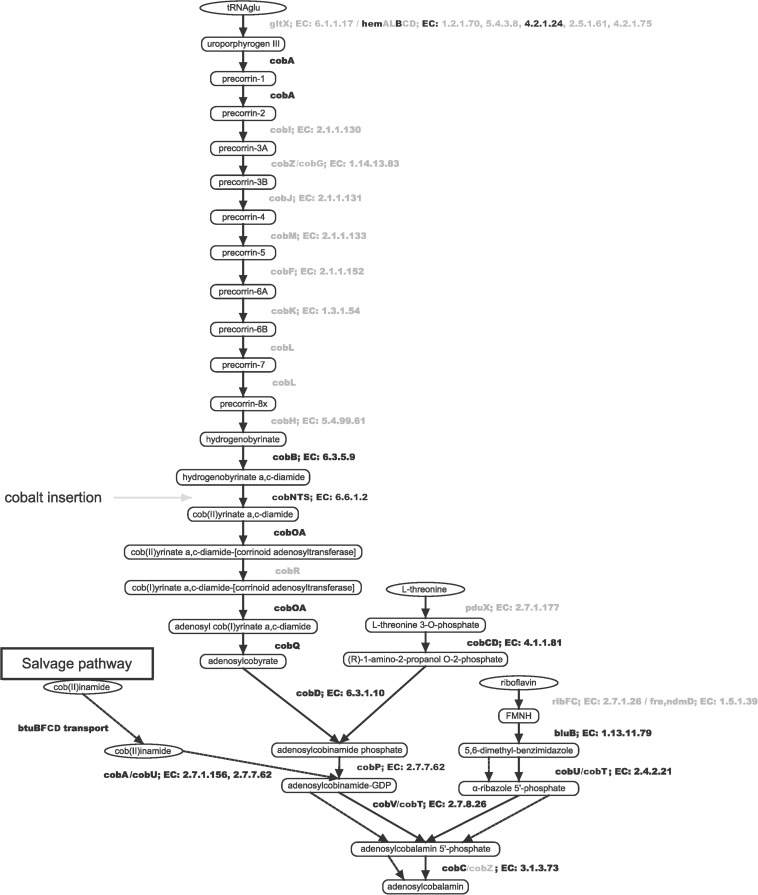
Reconstruction of the biosynthetic pathway of B12 in *S*. *haloaromaticamans* based on its genome analysis and assemblage with the Pathway Tools software suit v18.5 (Karp et al., 2015). Enzyme names indicated with black or gray color were either detected or not detected in the genome of *S. haloaromaticamans*, respectively. Dashed arrows denote the steps of the cobalamin salvage pathway. Gene annotation evidence according to BioCyc and RefSeq database hits is provided in Supplementary Table S2.

As a next step we reconstructed the biosynthetic pathway of B12 in *S. haloaromaticamans* to investigate its potency to synthesize the co-factor. The bacterial genome lacked all the genes for the biosynthesis of the corrin ring of B12 (*cobIZGJMFKLH*) (Figure 3 and Supplementary Table S2). Instead it carries nearly all genes of the lower pathway (*cobABNSTWOCQDPUVCZ*) from the conversion of hydrogenobyrinate to its a,c-diamide derivative through Co^2+^ chelation to adenosyl-cobalamin (Raux et al., 2000).

The absence of a *de novo* biosynthetic pathway of B12 in *S. haloaromaticamans* genome and its demonstrated absolute requirement for B12-dependent methionine synthesis, led us to speculate that the bacterium should possess a high affinity system for uptake and translocation of corrinoids. The bacterial genome was searched for genes encoding for energy-dependent uptake of B12. We detected 88 genes with potential role in the energy-dependent uptake of various biomolecules including B12: 43 were annotated to code for TonB-dependent receptors (outer membrane transportation system), 20 coded for the TonB/ExpB/ExpD complex components (inner membrane transportation system), 21 genes coded for genes with a potential role in the regulatory system of the TonB-dependent uptake (20 comprising the FecI/FecRand one being the iron uptake repressor “fur” gene), and 4 genes were screened due to either their vicinity to TBDT or potential association (due to their annotation or their content) with riboswitches (Supplementary Table S3). *Sphingomonadales* genomes are known to be rich in TBDT [i.e., *Sphingomonas wittichii* RW1 carried 134 TBDTs (Tang et al., 2012)], in line with their high abundance in the genome of *S. haloaromaticamans*. From the 43 TonB-dependent receptors (i) 16 were annotated as putative B12 outer membrane receptors (BtuB) (Chimento et al., 2003), showing best sequence matches to the previously published *S. haloaromaticamans* strain A175 DSM13477 genome (Wittich et al., 2007) (ii) seven were annotated as colicin receptors (CirA) (Ferguson et al., 2002), and (iii) the rest as ferripyoverdine (FpvA), ferric pseudobactin (PupA) (Adams et al., 2006), ferrichrome iron (FhuA) (Locher et al., 1998) and various siderophore receptors (Noinaj et al., 2010). Considering the very close structures of the several TBDT, including B12/colicin/siderophore transporters, their sequence-based substrate determination should be considered with caution (Koster, 2001; Rodionov et al., 2003).

**FIGURE 4:**
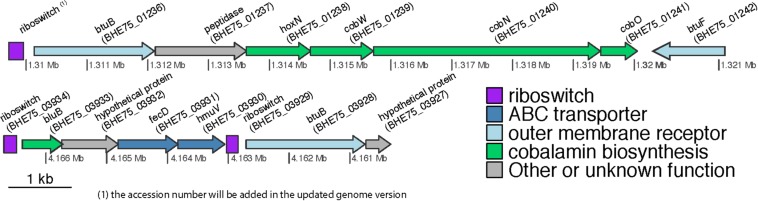
Genetic organization of the genomic regions in *S. haloaromaticamans* downstream of the three riboswitches associated with B12-biosynthesis. Genes annotation *btuB*: TonB-dependent corrinoid transporter; *hoxN*, high affinity Co^+2^/Ni^+2^ transporter; *cobW*, cobalt-chelation associated protein; *cobN*, cobalt chelatase subunit N; *cobO*, corrinoid adenosyltransferase; *btuF*, substrate-binding component of the ABC cobalamin transporter system; *bluB*, 5,6-dimethylbenzimidazole synthase; *fecD*, permease of a *btuCD*-like ABC cobalamin transporter system; *hmuV*, ATP-binding subunit of a *butCD*-like ABC cobalamin transporter system.

From these TonB-dependent receptors only three fulfill the stringent criterium of Degnan et al. (2014) in order to be assigned a cobalamin transporter function, “*to be located downstream of riboswitches*.” These are RNA elements that change conformation upon binding of a specific molecule affecting negatively or positively the transcription and translation of downstream genes including B12 uptake and biosynthesis (Montange and Batey, 2008). *In silico* screening of the genome for regulatory riboswitches identified three of them residing in the 5′ region of TBDT (Figure 4). The first (position of putative riboswitch: 1309712-1309955) was located upstream of a region encoding BtuB, a protein of the HoxN family known to encode high affinity Co^+2^/Ni^+2^ transporter genes (Komeda et al., 1997), *cobW*, *cobN*, and *cobO*, respectively, encoding proteins involved in Co chelation, cobalt chelatase subunit N and a corrinoid adenosyltransferase (Rodionov et al., 2003), all involved in the biosynthesis of B12, and BtuF, the substrate-binding component of the ABC transporter system for B12 uptake. Such a gene organization has been reported in several proteobacteria where *hoxN* and *cobW* are located immediately upstream of *cobN* and the whole locus is flanked upstream with a riboswitch (Rodionov et al., 2003). T he second (position 4162841-4163031) and the third riboswitches (position 4166483-4166691) were closely localized. The former is located upstream of *bluB*, a hypothetical protein and *fecD* (*e*-value1e-718, 78% identities and 86% positives with the *Sphingopyxis granuli btuC*, AMG76476.1), *hmuV* (*e*-value8e-97, 66% identities and 79% positives with the *Sphingopyxis* sp. strain C-1 *btuD*, GAO77867.1) genes encoding, respectively, a permease and an ATP-binding protein forming a *btuCD*-like ABC transporter component of the cobalamin transportation system. BluB belongs to the flavin destructase family proteins and controls the transformation of FMN_2_ to 5,6-dimethylbenzimidazole (Taga et al., 2007), which is the lower axial ligand of B12, and the preferred ligand in the cobamides of most bacteria (Hazra et al., 2013). Considering the key role of *bluB* in the biosynthesis or remodeling of salvaged corrinoids by bacteria it is not surprising that its translation might be regulated by B12-associated riboswitches. The third riboswitch is located in the 5′ region of *btuB*. In all other cases no riboswitches or other regulatory systems were detected. This could be due to missing alignment profiles from the databases which are necessary for identifying riboswitches in sphingomonads, or, given that cobalamin is a well-defined structure, maybe riboswitches do not exist as a regulatory mechanism. In the latter case, a different regulatory system or even a housekeeping role of the associated transporters could be possible.

Overall, our *in silico* analysis of the biosynthetic pathway of B12 suggests that *S. haloaromaticamans* is not able to biosynthesize B12 and meet its cellular needs for the methionine biosynthesis. However, its genome is enriched in receptors for transportation of corrinoids across its outer membrane, operating the energetically inexpensive salvage pathway to serve its needs in the primary cellular metabolism.

### B12 and Not Methionine Precursors Rescues the Degradation Capacity of *Sphingomonas haloaromaticamans*

**FIGURE 5:**
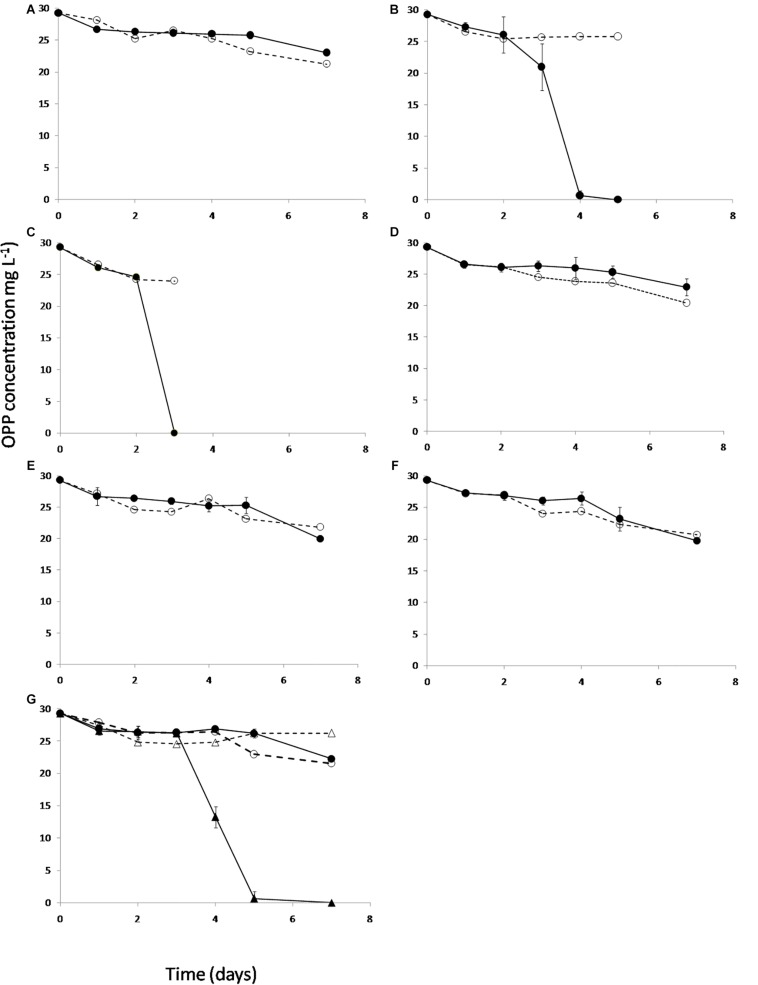
The degradation of OPP by *S. haloaromaticamans* in MSMN (•, solid lines) without any supplementation **(A)** and with supplementation of methionine **(B)**, B12 **(C)**, and of various intermediates of the methionine biosynthetic pathway like homoserine **(D)**, *O*-succinyl homoserine **(E)**, cystathionine **(F)**, and homocysteine [plus (▲) or minus B12 (•)] **(G)**. Each value is the mean of triplicates ± the standard deviation of the mean. In all treatments the degradation of OPP in non-inoculated cultures was also determined (∘, △ dashed lines).

Based on the genome sequence-based metabolic reconstruction evidence which suggested that *S. haloaromaticamans* was not capable of *de novo* biosynthesis of B12, we evaluated *in vitro* whether the supplementation of methionine precursors and/or B12 will rescue the degradation capacity of *S. haloaromaticamans*. The bacterium maintained its degrading capacity against OPP when supplemented with methionine (Figure 5B) and B12 (Figure 5C). However, it failed to degrade OPP when it was not supplemented with any of the above (Figure 5A) or supplemented with intermediates of methionine biosynthesis (Figures 5D–G) unless B12 was also co-provided as in the case of homocysteine (Figure 5G). These results verified genome-based metabolic reconstruction findings that B12 is the main driver of the auxotrophy of *S. haloaromaticamans* to methionine.

### The Degradation and Growth of *Sphingomonas haloaromaticamans* Supplied With a Range of B12 Concentrations

**FIGURE 6:**
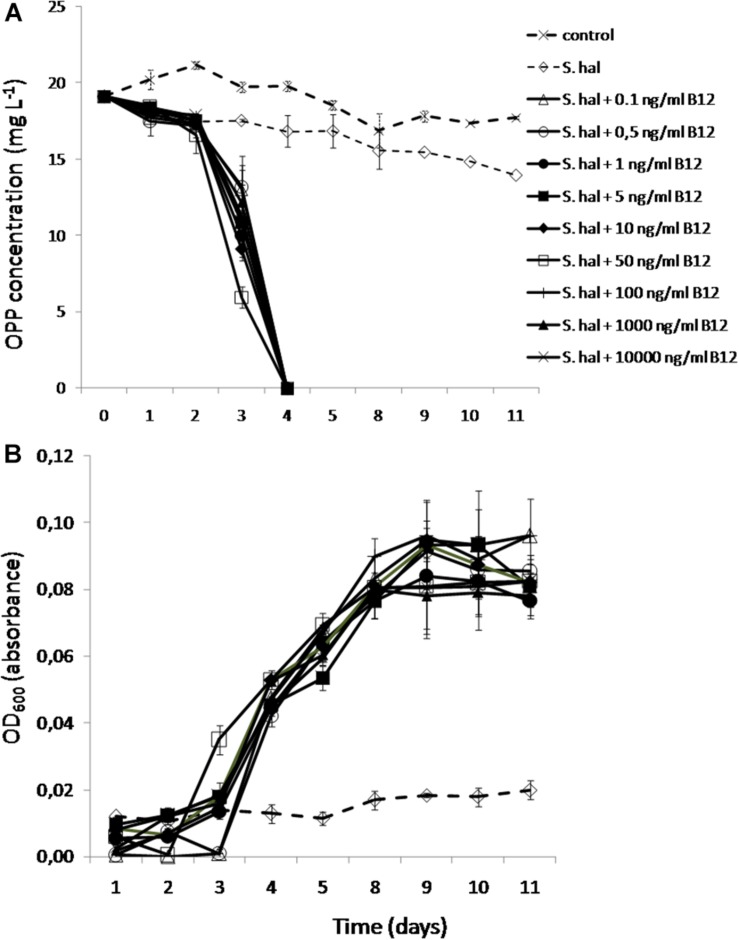
The degradation of OPP **(A)** and the growth of *S. haloaromaticamans* **(B)** in MSMN supplemented with 0, 0.1, 0.5, 1, 5, 10, 50, 100, 1000, and 10000 ng ml^–1^ of B12. The degradation of OPP in non-inoculated controls was also determined (control). Each value is the mean of triplicates ± the standard deviation.

We finally tested the minimum concentrations of B12 provided to *S. haloaromaticamans* to avert its auxotrophy to methionine and degrade OPP. The bacterium was able to degrade OPP even when supplied with 0.1 ng ml^–1^ of B12 (Figure 6A), which is close to the concentration levels of B12 and corrinoids found in fresh water (2–6 ng L^–1^) (Kurata, 1986), soil solution (5 μg L^–1^) (Mozafar and Oertli, 1992), and wastewaters (0.05–5.2 mg L^–1^) (Selimoglu et al., 2015). In addition, all concentrations of B12 supported similar growth rates of *S. haloaromaticamans* (Figure 6B). As expected, no growth and degradation of OPP was observed when the bacterium was not supplemented with B12.

## Conclusion

A combination of genome-based metabolic reconstruction and *in vitro* tests demonstrated that the auxotrophy of a fungicide-degrading *S. haloaromaticamans* strain was driven by the lack of a MetE B12-independent cobalamine synthase and the absence of a *de novo* B12 biosynthetic pathway. The bacterium could maintain its degradation and growth capacity in the presence of environmentally relevant concentrations of B12 suggesting that its application in natural and engineered systems would not be hampered by its auxotrophy to B12, in line with the presence in its genome of several potential B12 outer membrane receptors and a near complete cobalamine salvage pathway. Further studies will focus on (a) the corrinoids preferences of *S. haloaromaticamans* for optimum growth and OPP degradation using cross-feeding studies with corrinoid-producing bacteria (*Mesorhizobium loti*) and external supply of selected corrinoids (b) the role of the riboswitches detected on the regulation of cobalamin biosynthesis and corrinoids uptake.

## Data Availability Statement

The datasets generated for this study can be found in the DDBJ/ENA/GenBank accession number MIPT00000000.

## Author Contributions

CP isolated the microorganism, performed the *in vitro* experiment, and drafted the manuscript. SV performed the bioinformatic analysis and genome-based reconstruction, and helped in drafting of the manuscript. EP performed the *in vitro* experiment and reviewed the manuscript. DK had the experimental idea, supervised and planned the experiments, and revised the final manuscript.

## Conflict of Interest

The authors declare that the research was conducted in the absence of any commercial or financial relationships that could be construed as a potential conflict of interest.

## Supplementary Material

FIGURE S1 Phylogenetic analysis of the metB/Z gene encoding cystathione γ-synthase/O-succinyl homoserine sulfhydrylase detected in the genome of *Sphingomonas haloaromaticamans*.

TABLE S1 The functional annotation of the genes participating the biosynthetic pathways of methionine, isoleucine, tyrosine, and phenylalanine in the genome of the *Sphingomonas haloaromaticamans* strain. Annotation was performed using UniProt and RefSeq BLAST searches and verified further through Pfam searches.

TABLE S2 The functional annotation of the genes participating in cobalamin biosynthetic pathways in the *Sphingomonas haloaromaticamans* strain. Annotation was performed using UniProt and RefSeq BLAST searches and verified further through Pfam searches.

TABLE S3 The functional annotation of the genes associated directly or indirectly with the energy-dependent uptake of various biomolecules (i.e., cobinamides, siderophores, colicin etc.). Annotation was performed using UniProt and RefSeq BLAST searches and verified further through Pfam searches. Purple color denotes genes with TonB-dependent transporter function, orange color denotes genes with energy transducing role (ABC transporters), blue color denotes genes with regulatory function in B12 transportation and gray color denotes other genes with indirect association with B12 biosynthesis or transportation.
