# Supplementary figures and images for: Genome-Based Metabolic Reconstruction Unravels the Key Role of B12 in Methionine Auxotrophy of an Ortho-Phenylphenol-Degrading Sphingomonas haloaromaticamans
Source: Front Microbiol. 2020 Jan 10;10:3009. doi: 10.3389/fmicb.2019.03009 (PMC6970198; doi:10.3389/fmicb.2019.03009)

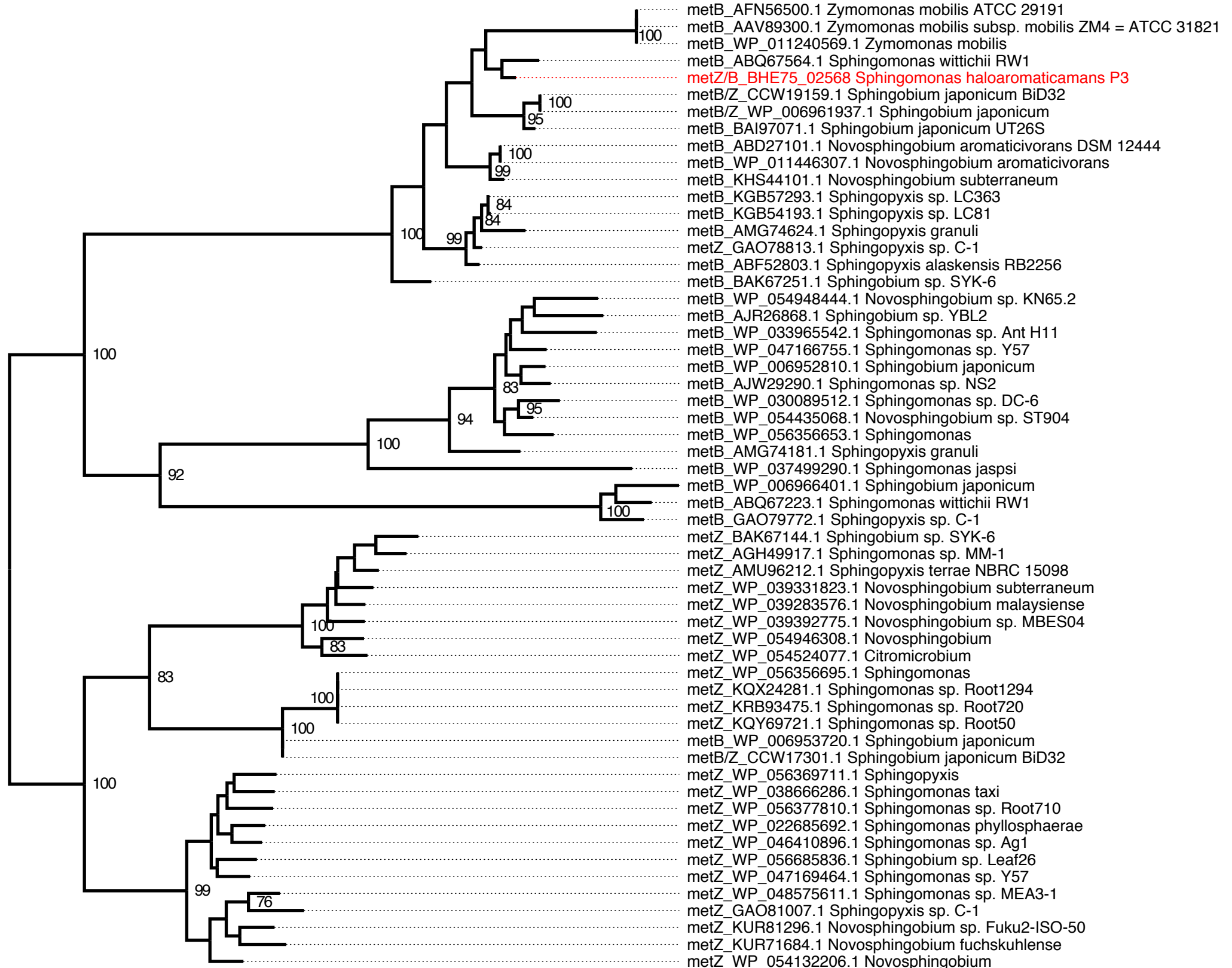

Supplement: FIGURE S1 — Phylogenetic analysis of the metB/Z gene encoding cystathione γ-synthase/O-succinyl homoserine sulfhydrylase detected in the genome of Sphingomonas haloaromaticamans. [file Data_Sheet_1.PDF]
